# Morphology controls how hippocampal CA1 pyramidal neuron responds to uniform electric fields: a biophysical modeling study

**DOI:** 10.1038/s41598-017-03547-6

**Published:** 2017-06-12

**Authors:** Guo-Sheng Yi, Jiang Wang, Bin Deng, Xi-Le Wei

**Affiliations:** School of Electrical and Information Engineering, Tianjin University, Tianjin, 300072 China

## Abstract

Responses of different neurons to electric field (EF) are highly variable, which depends on intrinsic properties of cell type. Here we use multi-compartmental biophysical models to investigate how morphologic features affect EF-induced responses in hippocampal CA1 pyramidal neurons. We find that the basic morphologies of neuronal elements, including diameter, length, bend, branch, and axon terminals, are all correlated with somatic depolarization through altering the current sources or sinks created by applied field. Varying them alters the EF threshold for triggering action potentials (APs), and then determines cell sensitivity to suprathreshold field. Introducing excitatory postsynaptic potential increases cell excitability and reduces morphology-dependent EF firing threshold. It is also shown that applying identical subthreshold EF results in distinct polarizations on cell membrane with different realistic morphologies. These findings shed light on the crucial role of morphologies in determining field-induced neural response from the point of view of biophysical models. The predictions are conducive to better understanding the variability in modulatory effects of EF stimulation at the cellular level, which could also aid the interpretations of how applied fields activate central nervous system neurons and affect relevant circuits.

## Introduction

The central nervous system (CNS) has powerful information processing capability, which is mainly responsible for relaying messages about muscle movement and sensory inputs. Nowadays, stimulating CNS with a dose of electric or magnetic field has become a common tool for probing brain physiology and cognitive function^1–6^. Such kind of technique includes transcranial magnetic stimulation (TMS) and transcranial direct current stimulation (tDCS)^1, 2, 4^. Due to their noninvasive interactions with brain tissue, they have been widely used for treating neuropsychiatric disorders in clinic and interfering with cognitive tasks^1–5^. However, the applications of these techniques have outpaced our scientific interpretations of their dose-response. The underlying mechanism of how they activate CNS neurons and affect relevant circuits remains elusive.

It has been shown that the electromagnetic fields applied affect CNS by generating a distributed electric field (EF) around the brain tissue underneath^5, 6^. The generated field results in the polarization of the membrane of nearby cells^7–9^. To be specific, neuronal elements are hyperpolarized near anode and depolarized near cathode. The spatial distribution of such polarization is not only governed by EF dose (including intensity, waveform, frequency, duration, orientation), but also dependent on cell biophysics and morphologies^7, 8, 10–12^. These factors make cellular response to applied EFs highly variable among neurons, which is difficult to precisely predict. In particular, *in vitro* studies^7, 8, 12^ have shown that cell morphology is a predominant factor for contributing to the variability in neural response to EFs. Such variable dose-response at the cellular level is a main barrier that prevents us to acquire new knowledge about the mechanical understanding of EF stimulation. It also restrains our deep interpretations of how EF dose affects large-scale network dynamics, CNS activity, cognitive ability, as well as ultimate behavior.

The principle carrier of information in a neuron is the sequences of action potentials (APs), which are usually initiated in the axon initial segment^13^. Such electrical impulses transmit input signals from one cell to the dendrite of others via synapses. When applying a dose of EF to a neuron, its dendrite, soma and axon are stimulated simultaneously. The complex structure and biophysics of these segments make the membrane at different sites have distinct degrees of hyperpolarization or depolarization. *In vitro* studies have shown that the distributed polarization induced by applied fields is able to give rise to various impacts on neuronal activity, such as altering polarity and amplitude of transmembrane potential^14, 15^, affecting kinetics of ionic channels^6, 12^, modulating epileptiform activity^16, 17^, regulating spike timing, firing rate or AP threshold^7, 8, 18, 19^, suppressing dendritic Ca^2+^ activity^20, 21^, and promoting bursting behavior^7^. However, it is still unclear how the morphologies of soma, axon or dendrites participate in the activation and response of single neuron induced by EF.

A crucial step toward addressing this question is to use computational models to describe cell morphologies and quantify their effects on field-induced responses. Until now, various models have been developed to interpret the modulatory effects of field stimulation. The point-neuron model is one commonly used type among them, ranging from Hindmarsh-Rose^22, 23^ and Fitzhugh-Nagumo^24^ models to multi-compartmental biophysical^25–29^ models based on Hodgkin-Huxley theory. Such simple models are amenable to both mechanistic analysis and efficient simulation, but the complex structures of realistic cell are unavailable in them. To effectively capture cell morphologies and biophysics, cable equation is adopted to simulate field-induced response^30–34^. They come to a similar conclusion that field-induced polarization is governed by morphological and biophysical details of membrane as well as temporal property of applied fields. But how cell morphology contributes to variable response of neurons to EF is still not well understood.

Unlike them, the current study focuses on hippocampal CA1 pyramidal cells. This type of neuron is an important cellular target of EF stimulation^35^, due to that their slender structure and parallel distribution are conducive to field-induced polarizations. We consider both subthreshold and suprathreshold stimulation here. Our goal is to uncover how basic and realistic morphologies of CA1 pyramidal cells participate in EF-induced polarizations and responses. To achieve this goal, we adopt biophysical models to describe cell morphology and quantify its relationship with excitation threshold required to activate neuron (i.e., rheobase). Such rheobase is also referred to as EF firing threshold, which is determined by stepwise increasing field intensity until an AP is initiated. This measure reflects the specific sensitivity of a neuron to suprathreshold fields, and its value depends upon the biophysics and morphologies of pyramidal cells relative to EF^7, 12, 27, 33, 34^.

## Results

### Responses of artificial neuron with specific morphology

Our starting neuron is an artificial model (see Methods) with simple two-dimensional spatial structure built in NEURON simulation environment. Although it is simplified, such artificial model allows us to capture the basic morphologies commonly found in CA1 pyramidal cells. By using NEURON extracellular mechanism^36^, we introduce uniform EF stimulus to artificial model. We first describe the firing properties of the neuron with specific morphology to EFs. Unless otherwise stated, the midpoint of soma is selected as the recording point.

**Figure 1:**
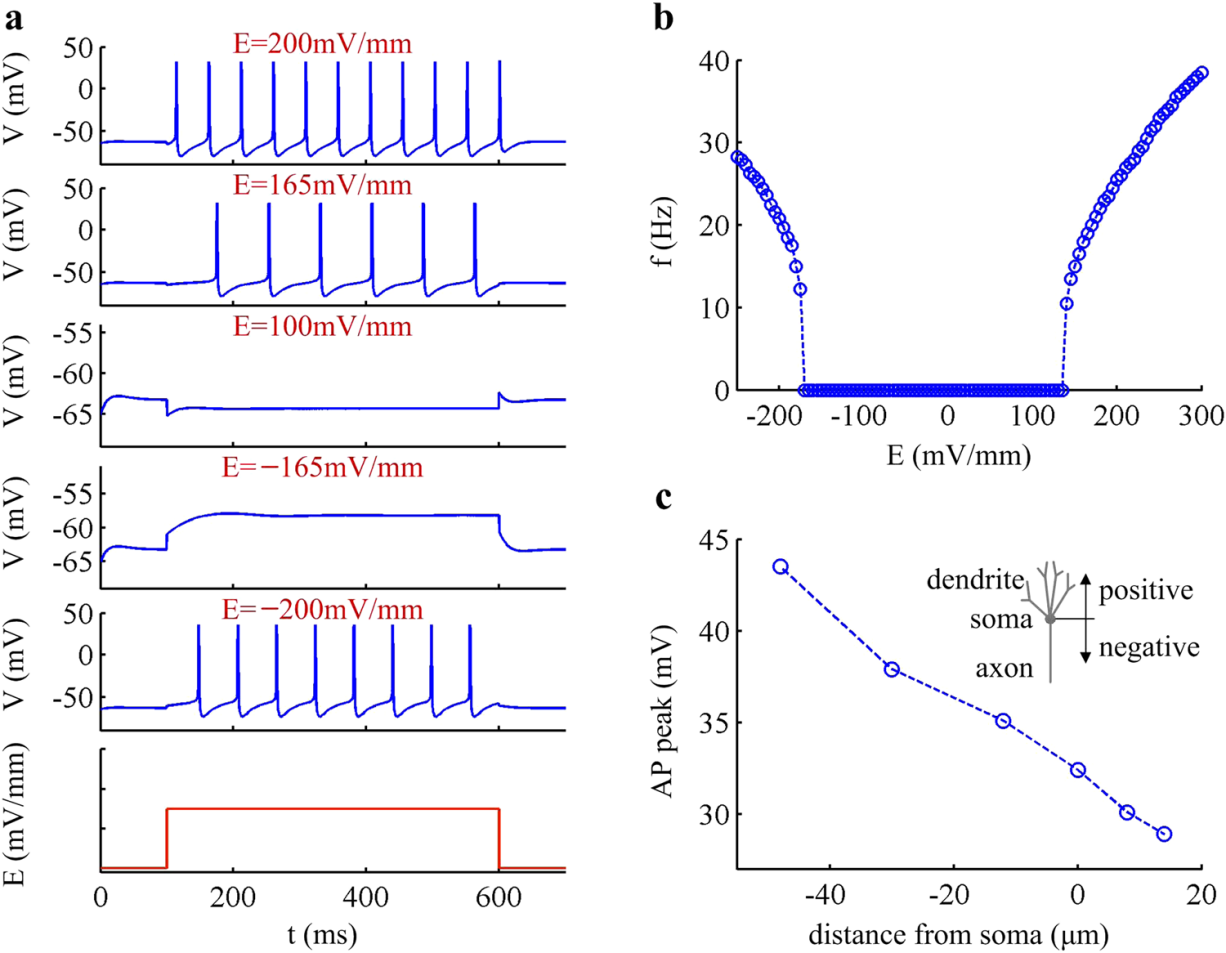
Responses of artificial neuron with specific morphology to EFs. (**a**) Typical spike trains recorded in the midpoint of the soma with several values of EF, which are indicated on the top of relevant panel. Bottom panel is used to indicate the time of the onset and offset of applied field. (**b**) Relationship between somatic firing rate and EF, i.e., *f*-*E* curve. (**c**) Peak value of the AP recorded at different sites of the artificial model. Relevant morphological parameters of soma, axon and dendrites take their initial value.

Figure 1a shows typical spike trains recorded in the midpoint of soma with different values of field *E*. One can observe that artificfial neuron is unable to initiate APs when EF does not have a sufficient intensity. Such weak fields are usually referred to as subthreshold stimulus. Once *E* exceeds a threshold voltage, both positive and negative EFs are able to trigger APs in artificial neuron. The ability of either polarity of EF to induce spiking could be interpreted by considering the effective current sources and sinks created by uniform field. We define the source as causing the flow of current (positive charges) toward the soma, and the sink as causing the flow of current away from the soma. Such current sources and sinks can be analogous to anterograde and retrograde APs. The sources contribute to depolarizing membrane while sinks result in the hyperpolarization of membrane. For positive EFs, the tips and branch points of the dendrites act as sources and the tip of the axon is a sink. The current sources and sinks reverse for the opposite polarity of EF. Since there are always current sources generated with both positive and negative fields, the artificial neuron is able to initiate APs once its membrane potential is forced to across a threshold level.

Here the EF firing threshold is respectively −172.4 mV/mm and 163.4 mV/mm (Fig. 1b). It is asymmetric because of the different efficacy of dendritic branches versus axonal structure in conveying field-evoked currents to AP initiation zone. The average firing rate *f* increases with EF intensity once *E* exceeds rheobase (Fig. 1b). The *f*-*E* curve is discontinuous for both positive and negative fields, and artificial neuron fails to fire slowly in response to EFs. This is the firing pattern recorded at the midpoint of soma. Other segments generate similar responses in the observed range of EF. Only difference is that the peak value of AP varies as the recording site alters (Fig. 1c). Specifically, the APs recorded in axon near cathode have larger peak value, whereas those recorded in the soma or dendrites near anode have smaller peak. Such difference in AP peak is largely due to the inhomogeneity of conductance densities with respect to the distance from the soma. In following simulations, we systematically quantify the impacts of basic and realistic morphologies on neural response to positive EFs.

### Effects of varying diameter

The diameter of neuronal element is one common morphological factor in single cell. Varying diameter alters axial resistance and relevant current, and then modulates neural response to EFs. Here we vary compartment diameter of the soma, axon, or dendrites in artificial neuron, and respectively examine their effects on EF threshold for triggering APs.

**Figure 2:**
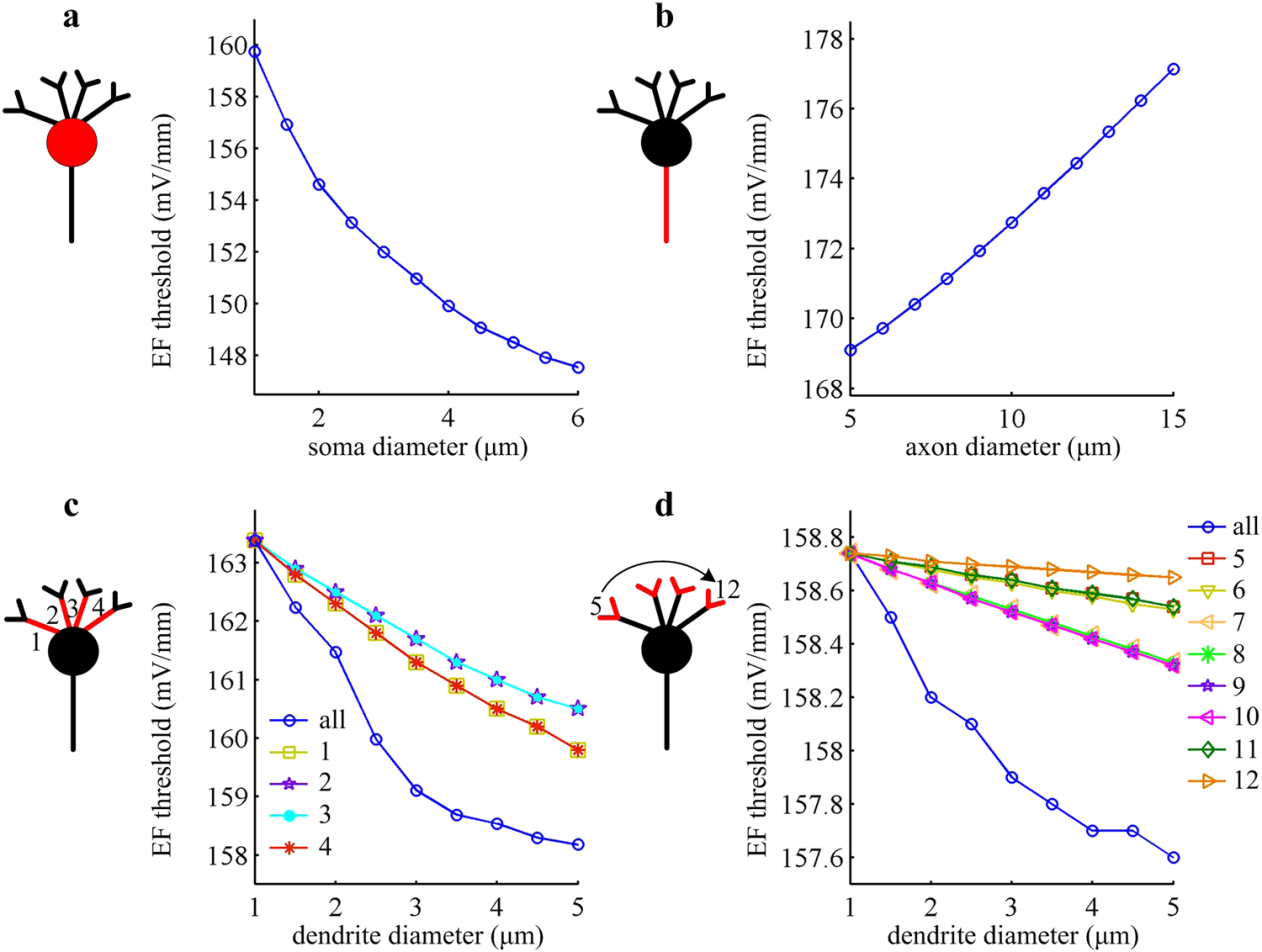
EF firing threshold changes with compartment diameter in artificial model. EF threshold for triggering APs is respectively showed as a function of the diameter of (**a**) soma, (**b**) axon, (**c**) proximal dendrites, and (**d**) distal dendrites. The length of relevant compartment takes the initial value.

The *f*-*E* curve recorded in the soma is always discontinuous when we vary the diameter of each segment in artificial neuron (data is not shown). But altering the diameter of soma, axon, or dendrites produces significant impacts on EF firing threshold. It is observed that EF threshold decreases as soma diameter is increased (Fig. 2a). It is known that increasing the diameter of soma decreases its axial resistance^36^. Since the EF remains constant, the difference between extracellular potentials of somatic compartment also remains constant. Under this condition, the axial current induced by EF increases with soma diameter, which produces larger depolarization on somatic membrane and results in a lower EF threshold. Contrary to soma, the EF threshold increases monotonically with axon diameter when the soma and dendrites are far from cathode (Fig. 2b). Similar to above, increasing axon diameter decreases its axial resistance. Since the difference in extracellular potential along the axon remains constant, the current sink created by leakage through the axon is increased, effectively hyperpolarizing the soma and increasing EF threshold.

Unlike axon diameter, the EF threshold for eliciting APs is inversely correlated with dendrite diameter (Fig. 2c and d). Increasing diameter reduces the axial resistance of relevant dendrites and leads to more effective sources. Then, a larger depolarization in soma is caused for a positive field. Accordingly, there is a decrease in EF threshold for the manipulations shown in Fig. 2c and d. For symmetric dendrites with respect to central axial, varying their diameter alone produces identical impacts on EF firing threshold. Simultaneously, varying the diameter of proximal dendrites leads to more significant decrease in EF threshold than distal dendrites. This is because the passive properties of dendrites filter and attenuate distal current signals as they spread to soma^37, 38^. Such passive integration by dendrites results in a progressively smaller somatic depolarization^38^, and thus a smaller impact on firing behavior. In addition, varying the diameter of several dendritic segments simultaneously causes more effective sources and decreases EF threshold, although its effects are not linear superposition of the cases of changing one alone. These simulations indicate that varying the diameter of soma, axon or dendrites results in distinct variations in the sensitivity of artificial neuron to suprathreshold field stimulation.

### Effects of varying length

**Figure 3:**
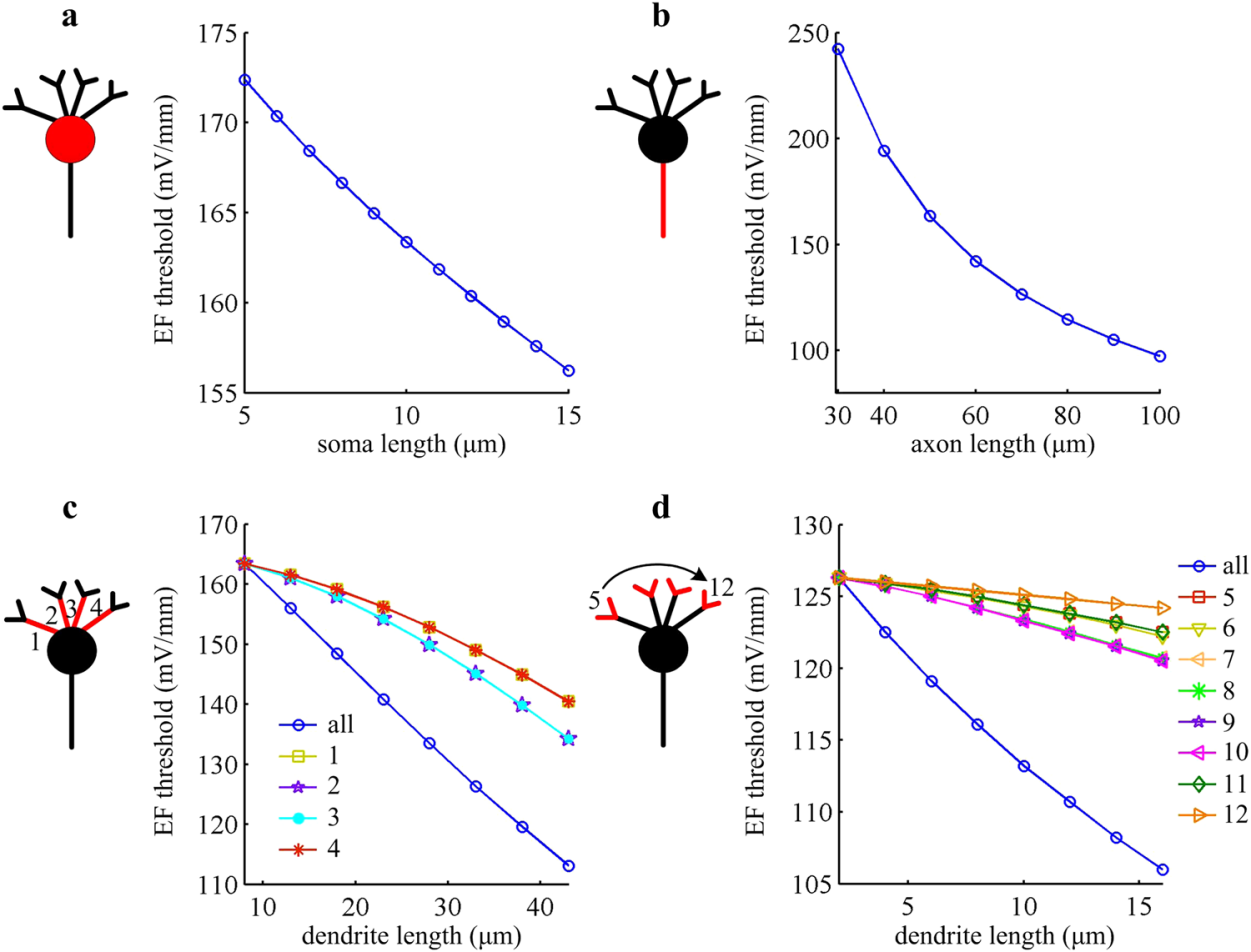
EF firing threshold negatively correlates with compartment length in artificial model. EF threshold for triggering APs is respectively showed as a function of the compartment length of **(a)** soma, **(b)** axon, **(c)** proximal dendrites, and **(d)** distal dendrites. The diameter of relevant compartment takes the initial value.

Apart from diameter, another basic morphology for neuronal element is the length. Figure 3 summarizes the effects of altering compartment length on EF-induced response in artificial neuron. Unlike varying diameter, increasing the length of soma, axon, or dendrite all leads to a decrease in EF firing threshold. According to cable theory, extending compartment length results in an increase in total axial resistance^36^. Such manipulation also increases the difference in extracellular potential to the degree that the increase in length is in vertical direction (see equation 4 in Methods). The net effect on current flow determines whether the efficacy of sinks or sources is improved. Unlike changing diameter, the net effect here is difficult to be identified. Even so, we could give some predictions about it according to the results shown in Fig. 3. For soma, the net effect of increasing its compartment length is to increase the axial current, which results in more depolarization on soma. Then, a lower EF threshold is required by artificial neuron to initiate APs (Fig. 3a). For axon, increasing compartment length reduces the efficacy of sinks, which leads to less hyperpolarization on soma and then decreases EF firing threshold (Fig. 3b). For dendrites, the net effect of increasing their length is to improve the efficacy of sources and decrease field threshold (Fig. 3c and d).

### Effects of dendrite structure

The dendrite in a realistic cell may branch multiple times, which gives rise to a complex dendritic tree. Such tree-like structure makes the dendrites act like antennas receiving information from thousands of other cell, sometimes tens or even hundreds of thousands. The complex structure of the dendritic tree has been shown to play a crucial role in both AP generation and neural coding^37, 38^.

**Figure 4:**
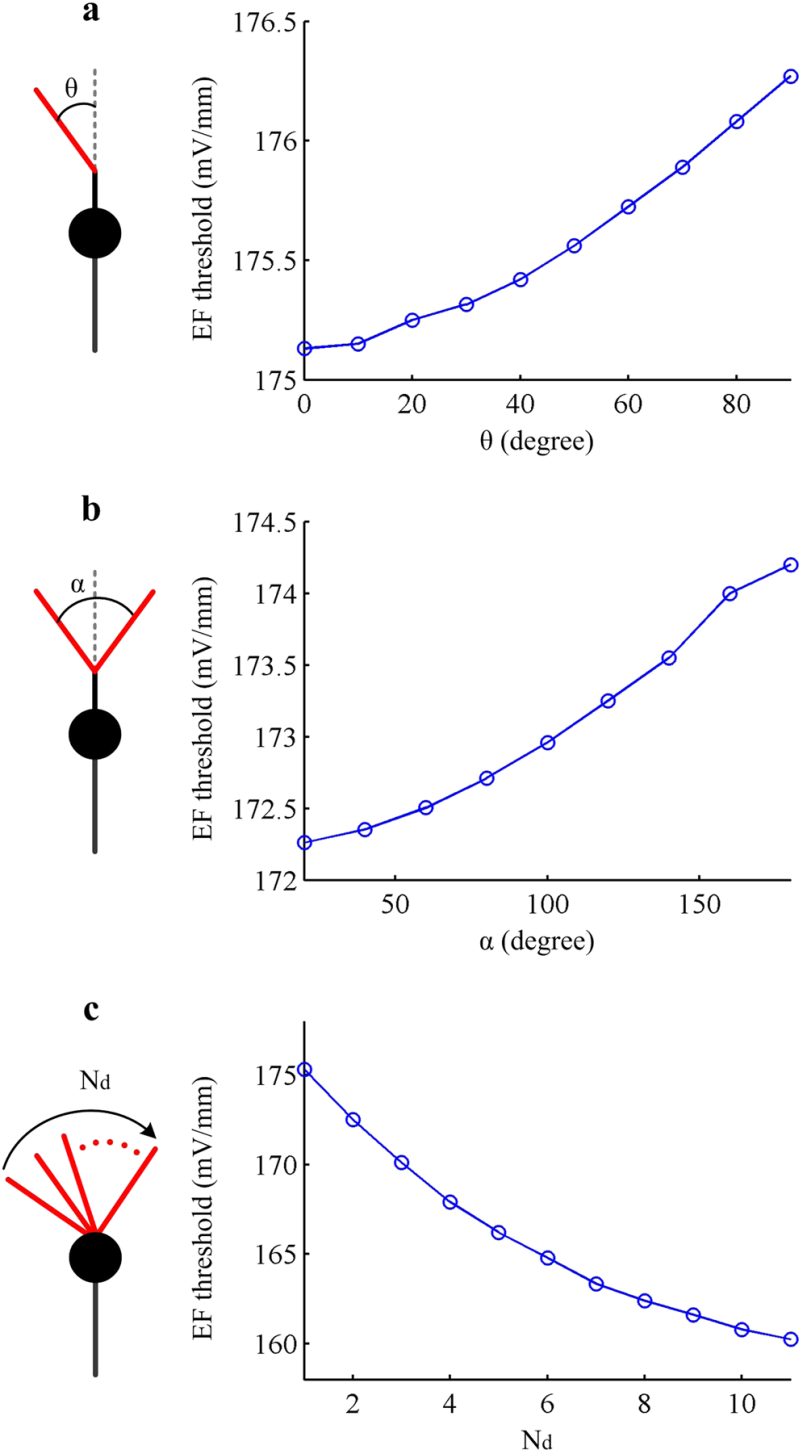
Basic morphologies of dendrites affect EF firing threshold in artificial model. EF threshold for evoking APs is respectively showed as a function of **(a)** bend angle β, **(b)** bifurcation angle α, and **(c)** branch number N_d_ of the dendrite. The diameter and length of each dendrite are 2 μm and 8 μm. Other morphological and biophysical parameters of the artificial neuron are as indicated in Methods.

Here we characterize how the basic structures of dendrites affect the response of artificial neuron to suprathreshold fields. We observe that the EF threshold for eliciting APs in soma increases with the angle of bend or branch (Fig. 4a and b). In contrary, increasing dendrite number reduces EF firing threshold, and there is an inverse correlation between them (Fig. 4c). These phenomena are easy to interpret. The bend or branch angle greater than 0 from the vertical direction decreases the gradient of field-induced extracellular potential along the dendrite, which reduces its effectiveness as a source and then raises EF firing threshold. Adding dendritic branches in artificial neuron improves the efficacy of current sources and then decreases EF threshold.

**Figure 5:**
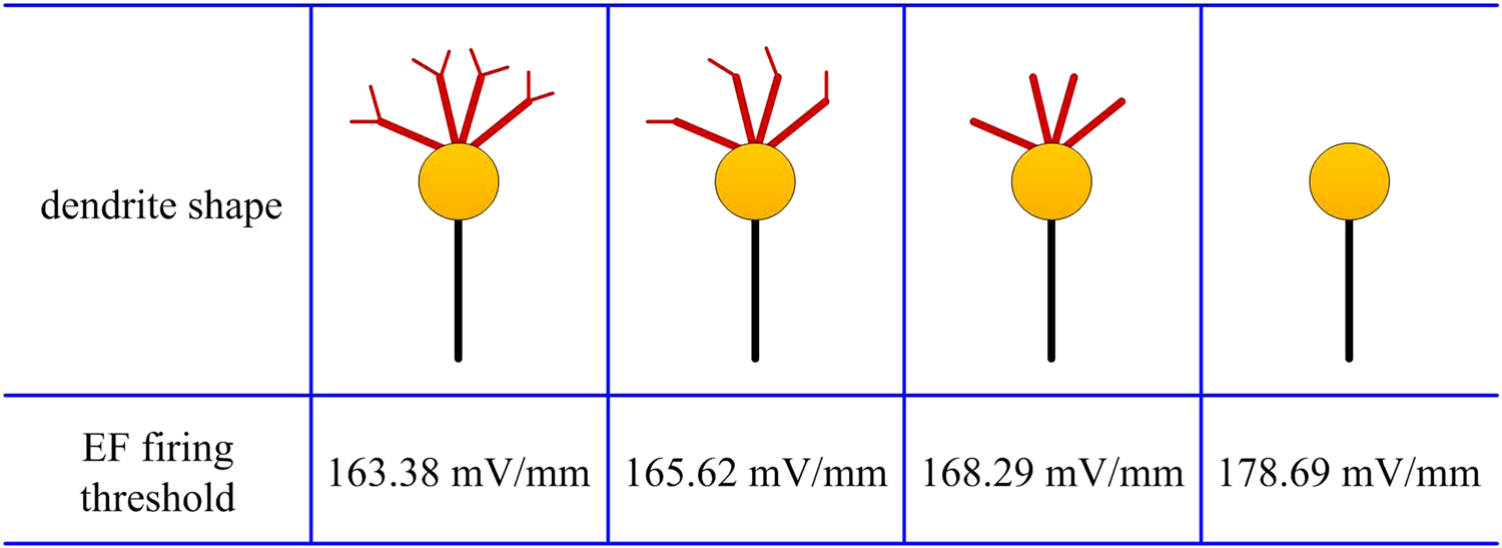
Dendrite structures affect EF firing threshold in artificial neuron.

We have also successively removed several dendritic branches from artificial neuron according to their distance from soma, and then determined its effects on field-induced response. From Fig. 5, one can find that such manipulations lead to a higher EF firing threshold from left to right. In fact, removing dendrite branches could be regard as a case of reducing current sources, thus decreasing effective sources and increasing EF threshold for triggering APs.

### Effects of axon structure

**Figure 6:**
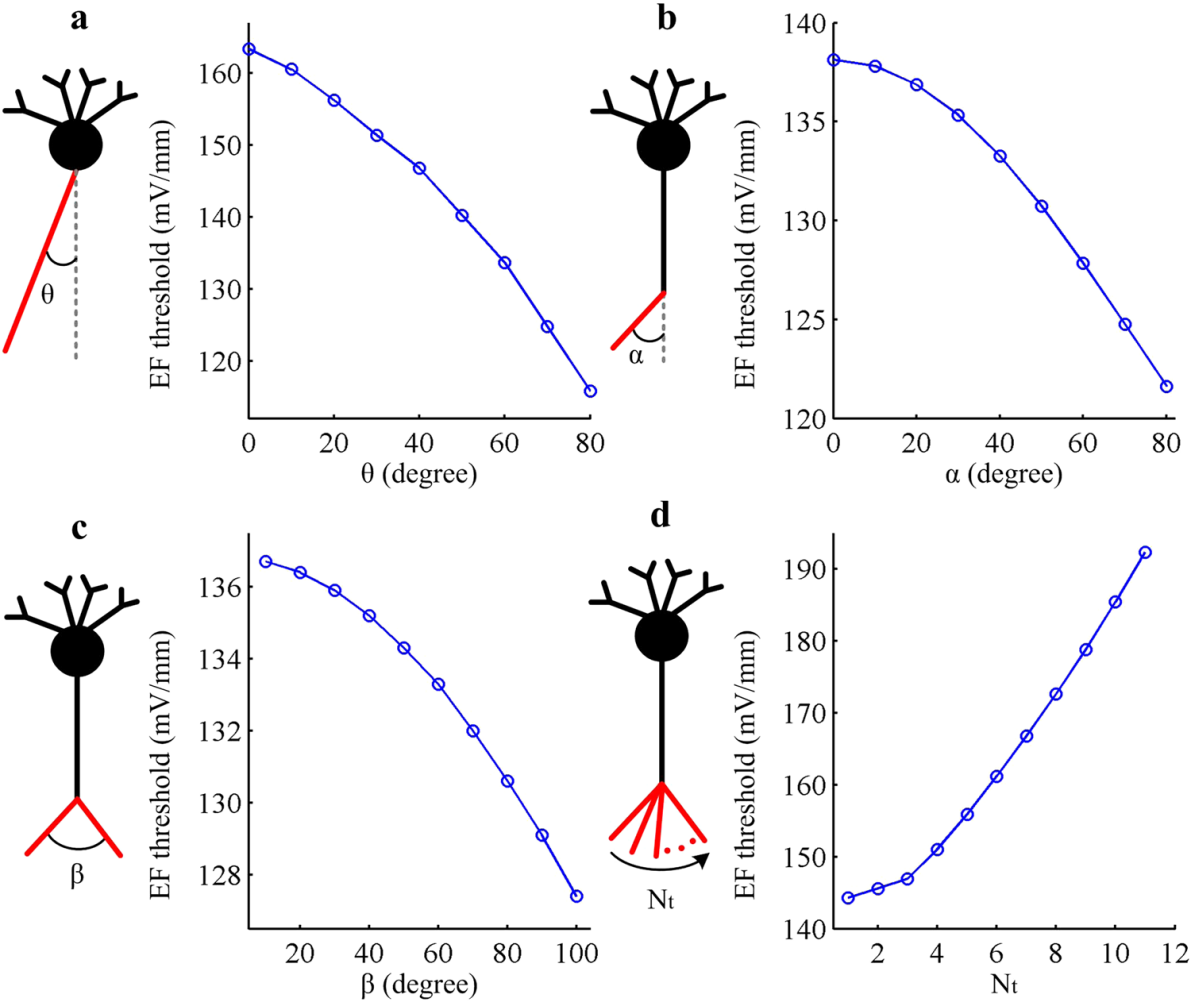
Basic morphologies of axon affect EF firing threshold in artificial model. **(a)** and **(b)** give the EF threshold as a function of two types of bend angle θ and α. **(c)** and **(d)** show the field threshold as a function of branch angle β and branch number N_t_ of axon terminal. The diameter and length of each branch (indicated by red lines) in **(b–d)** are 2 μm and 20 μm, respectively. Other morphological and biophysical parameters of the artificial neuron are as described in Methods.

In realistic neuron, there may be several branches at the end of axon, which allows the cell to connect with more than one targeted dendrite. Previous studies^6, 31, 33, 34^ have shown that the axon terminals play a crucial role in determining field-induced polarizations and APs, especially their bend or bifurcation as they enter the white matter. Here we introduce two kinds of bend as well as a bifurcation in the axon of artificial neuron. It is observed that increasing bend angle between axon and vertical axis or bifurcation angle between two terminal branches both results in a reduced EF firing threshold (Fig. 6a–c). In fact, introducing such bend or bifurcation decreases the gradient of extracellular potential along the axon while produces no effects on axial resistance. Then, all of these manipulations reduce the axon effectiveness as a sink and further decrease EF threshold. Besides, we have also investigated how varying the number of axon terminal affects neural response to uniform EF, which is shown in Fig. 6d. Increasing terminal number is to add the current sinks, which effectively hyperpolarizes soma and increases EF firing threshold. As expected, these simulations suggest that the spatial structure of axon terminal indeed participates in field-induced cellular response, which are comparable to earlier modeling^31, 33, 34^ and experimental^39, 40^ studies.

### Effects of introducing excitatory postsynaptic potential

Various spatiotemporal patterns of signals are transmitted through the synapse between two cells *in vivo*. When active presynaptic cell releases neurotransmitters into excitatory synapse, it results in the flow of positive ions into postsynaptic cell and generates an excitatory postsynaptic current (EPSC). The EPSC depolarizes the membrane of postsynaptic cell, and then excitatory postsynaptic potential (EPSP) is evoked^41^. The presence of such depolarizing potential increases the excitability level of postsynaptic cell and makes it easier to initiate APs^33, 41^.

**Figure 7:**
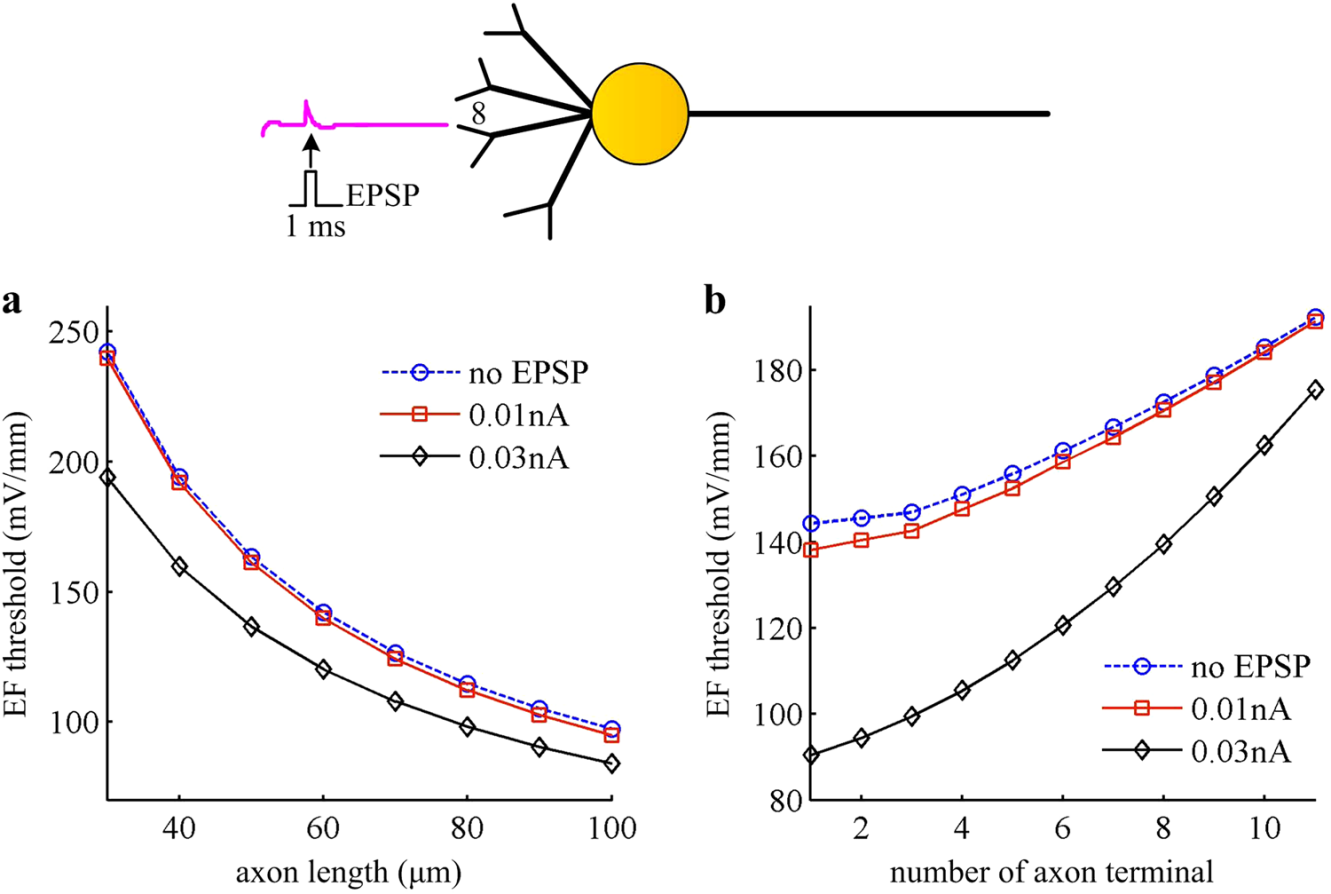
Introducing EPSP reduces EF firing threshold in artificial model. The EPSP is modeled as a weak current pulse ${I}_{{\rm{EPSP}}}$, and it is introduced to No. 8 distal dendrite. The relevant amplitude of ${I}_{{\rm{EPSP}}}$ is set to 0.01 nA and 0.03 nA. **(a)** and **(b)** respectively shows the EF firing threshold as a function of axon length and its terminal number in the cases of ${I}_{{\rm{EPSP}}}=0{\rm{nA}}$ (i.e., no EPSP), 0.01 nA, and 0.03 nA.

Here, we investigate how artificial neuron responds to EFs in the presence of EPSP, which is simulated by a subthreshold current pulse applied to No. 8 distal dendrite. We repeat the simulations in Figs 3b and 6d. As expected, introducing EPSP is indeed able to decrease the EF threshold for triggering APs (Fig. 7). The larger the EPSP, the larger will be the depolarization in soma for a specific positive field, resulting in a lower EF firing threshold. This indicates that artificial neuron becomes more likely to be activated by EFs in the presence of EPSP. Moreover, the field-induced firing threshold with EPSP is also controlled by cell morphologies. We only show the cases of altering axon length and its terminal number in Fig. 7. In fact, such predictions are applicable to other basic morphologies.

### Effects of realistic morphology

Previous sections have determined the influence of basic morphologies of an artificial neuron on its firing behaviors evoked by suprathreshold EFs. Here, we use three established multi-compartment models to examine how the cells with different realistic morphologies respond to subthreshold field. Three cells are respectively the CA1 pyramidal neuron in rat hippocampus, the L4 spiny neuron in cat visual cortex and the interneuron in rat dorsal lateral geniculate nucleus.

**Figure 8:**
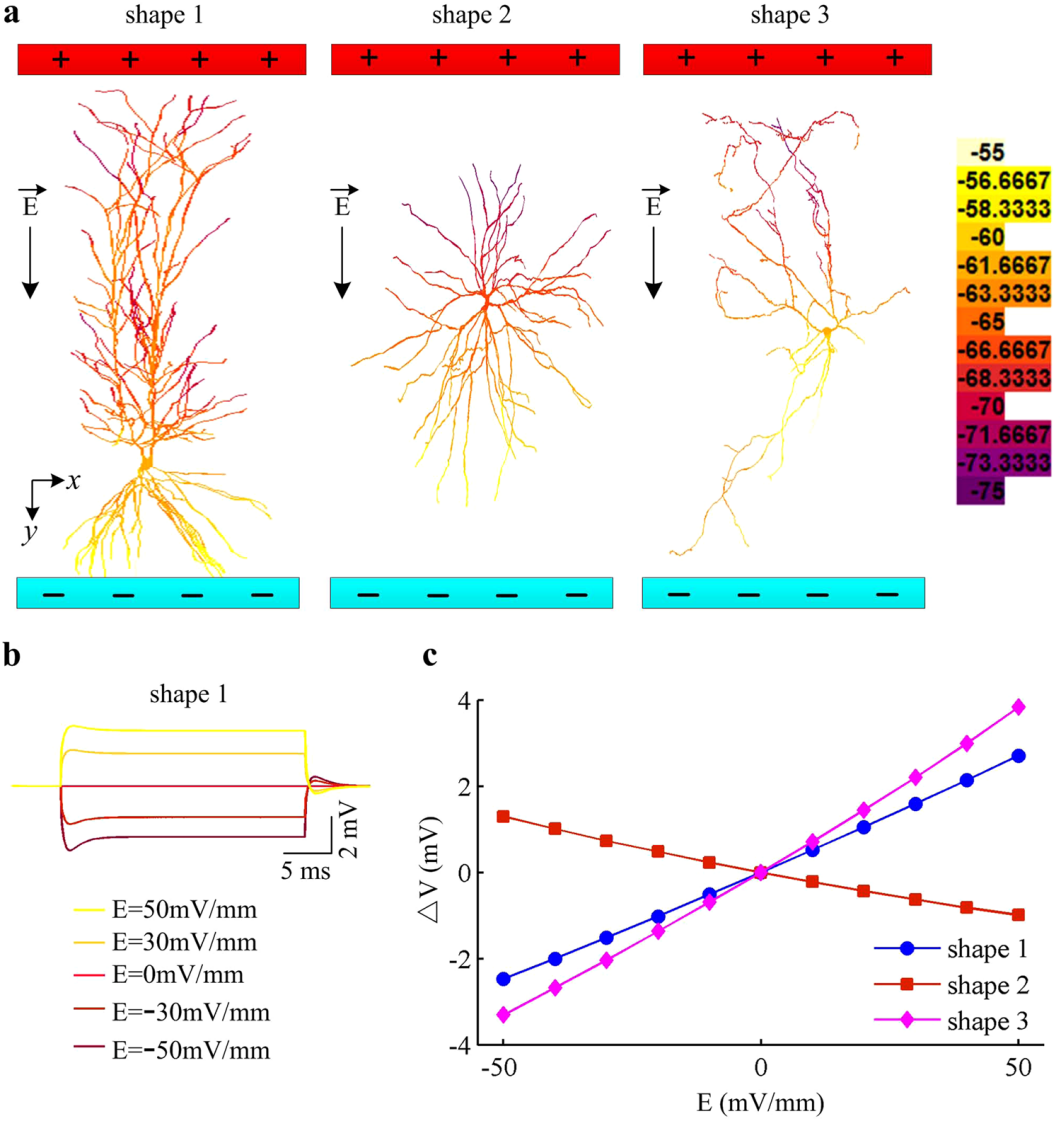
EF induces distinct polarizations in the cells with different realistic morphologies. (**a**) Polarization profiles of three established model neurons induced by subthreshold EF. They are respectively CA1 pyramidal neuron (shape 1), spiny neuron (shape 2), and interneuron (shape 3). The black arrow indicates the direction of applied EF and its intensity is 50 mV/mm. **(b)** Membrane voltage recorded at the midpoint of soma in rat CA1 pyramidal neuron with five values of EF. **(c)** Polarization profile at the center site of soma as a function of EF for three types of neurons.

Figure 8 shows the membrane polarization throughout each cell under the stimulation of weak EF. It is found that the cathode depolarizes cell membrane and the anode hyperpolarizes it. Such polarizations depend on the orientation of the dendrites relative to EF, and rotating the neuron within the field will change the polarity. This is true for all three realistic model neurons. Moreover, three neurons generate obviously distinct polarization profiles to subthreshold EF. Figure 8c shows the induced polarization profile of soma as a function of EF for each cell. One can observe that applying weak EFs to realistic model neuron linearly polarizes its soma membrane, and the polarization effect varies from cell to cell. Compared to spiny neuron, applied field induces opposite polarizations on the membrane potential of CA1 pyramidal neuron and interneuron. These simulations can also be accounted for in terms of the efficacy of sinks and sources. In both CA1 pyramidal neuron and interneuron, there are more dendrites near anode and less near cathode (Fig. 8a). Such asymmetrical distribution of dendrites in both cells makes them generate more sources and less sinks in the case of positive EFs. Then, the net effect of current flow is to depolarize soma. The larger the EF, the larger is the depolarization in soma. For negative EFs, the sources and sinks reverse, and in this case the net effect of current flow is to hyperpolarize soma. As a result, the polarization profiles in two kinds of neurons both increase in proportion to EF. On the contrary, the radial structure of spiny neuron makes less dendrite near anode and more near cathode, which leads to less sources and more sinks in the presence of positive fields. Then, the net effect of current flow is to hyperpolarized soma. Accordingly, the polarization profile in this cell is negatively correlated with EF intensity. These simulations suggest that morphological features play a crucial role in determining the spatial polarizations of realistic model neuron to subthreshold EFs.

## Discussion

We have presented an artificial model of CA1 pyramidal cell with simple spatial structure in NEURON environment. Numerical simulations show that the basic morphologies of soma, axon, or dendrites (including diameter, length, bend, bifurcation and branch number) are all capable of participating in EF-induced cellular response. Varying them nonlinearly alters EF threshold for triggering APs, and impacts cell sensitivity to suprathreshold fields. The results are interpreted by considering the effective sources and sinks caused by applied EFs, since their efficacy is directly related to cell excitability. The presence of EPSP conduces to the depolarization of soma and further decreases EF firing threshold. When such excitatory synaptic activity coincides with applied EFs, neural responses also depend upon cell morphology. Further, we have simulated the effects of subthreshold EFs on three realistic model neurons. It is found that the spatial polarizations induced by weak field are governed by realistic morphology. These numerical results highlight the significant role of morphological factors in determining field-induced cellular response.

Earlier experimental^7–9, 12, 14, 15^ and modeling^31–34^ studies have suggested that morphologic features govern the polarizations and firing behaviors of a neuron to electric or magnetic field stimulus. In particular, Radman *et al*.^7^ found that the cortical cells with different morphologies, i.e., L2/3 pyramidal neuron, fast spiking interneuron, and L4/5 pyramidal neuron, have different EF thresholds for AP generation; Pashut *et al*.^12, 33^ reported that magnetic field threshold for triggering spikes in L5 pyramidal neuron and interneuron is dependent on their morphology; Wu *et al*.^34^ recently showed that the tortuosity and arborization of axonal segment govern electromagnetic field threshold for eliciting APs in L5 pyramidal neuron. Unlike these existing observations, our modeling study has successfully identified how basic morphologies of CA1 pyramidal neuron participate in its activation triggered by EFs. This is not reported in previous physiological experiments or theoretical simulations. Our finding indicates that the factor of cell morphology is able to predict and explain the variability in EF-induced cellular response among cells. Note that different outcomes would be achieved if applied field is not uniform or cell biophysics and morphologies are changed.

In earlier studies^27–29^, we proposed a conductance-based model with two compartments to interpret the biophysical basis for AP initiation evoked by EFs. Our point-neuron model is consisted of soma and dendrite, which involves one geometrical parameter describing the relative area occupied by soma. A plausible prediction obtained with our point-neuron model is that geometrical feature controls AP initiating dynamics to EFs through altering the axial current between two compartments. Such prediction also applies to the results in present study. Introducing EF to a neuron with complex morphology causes perturbations on extracellular potential of neighbor compartments, which results in an additive axial current when it interacts with cytoplasmic resistor. This additive current is tightly related to cell morphologies and biophysics, which spreads to AP initiation zone and alters neural response to EFs. Unlike two-compartment model, it is infeasible to perform phase plane or bifurcation analysis in NEURON to describe in detail how APs are initiated. Even so, we have gained an interpretable understanding about the basic principles of how cell morphology influences EF-induced responses, which is unavailable with our point-neuron model.

As mentioned in Introduction, EF is a typical distributed stimulation for neural tissue. Contrary to it, current injection is a type of single-point stimulation, which has attracted broad attention in neurophysiology. According to frequency-current (*f*-*I*) curve, Hodgkin identified two major types of excitability^42^. Type I excitability corresponds to a continuous *f*-*I* curve, and Type II is discontinuous. Two types of excitability have been shown to represent distinct AP initiating dynamics^43, 44^, which arises from subthreshold interactions between inward and outward membrane currents. As for CA1 pyramidal cells, they can exhibit either Type I^45, 46^ or Type II^46–48^ excitability under current stimulus. In current study, the artificial neuron fails to fire slowly to suprathreshold EFs. According to our earlier findings with two-compartment model^27–29^, such abrupt threshold in *f*-*E* curve is generated through a Hopf bifurcation, consistent with Type II excitability. However, this prediction is based on *f*-*E* curve, not *f*-*I* curve or bifurcation structure of considered model, which makes it problematic. For the biophysically realistic models in present study, it needs future studies to determine its AP initiating dynamics to distributed field stimulation.

One central issue for interpreting neuromodulatory effects of magnetic or electric field stimulation is which element of targeted cells is most likely to be activated^33, 49^. The inhomogeneity of conductance densities throughout the cell endows different elements with distinct threshold of activation. Meanwhile, introducing distributed stimulus will result in different gradients of induced EF along the cell. These two factors codetermine the shape of membrane polarization, thus the field threshold for triggering APs and the initiation site of AP. Especially the latter may be the axon initial segment^49^, the location of maximal gradient of induced EF^33^, or where axons bend as they enter the white matter^31, 50^. For our artificial model, APs are always initiated at the axon initial segment during uniform EF stimulation, and then somatic APs are elicited since this site is closest to initial segment. Such prediction is comparable to earlier modeling studies of L3/5 pyramidal neurons^49^. Further, it has been shown that the EF threshold for activating cells is tightly related to field-induced physiological responses^49, 51^, including motor evoked potential, direct wave (D-wave) and indirect wave (I-wave). In this sense, we predict that variability in modulatory effects of TMS or tDCS can be attributed to the morphological distinctions of cell types.

The recording site of APs in our simulation is the soma, which results in a time delay as AP propagates from initiation zone to soma. As we know, the propagation speed of AP within a neuron is finite^52–54^, which is related to fiber diameter^54^. Then, varying compartment length or diameter also affects somatic AP latency. But this does not mean that the EF threshold for evoking APs in CA1 pyramidal model depends upon such latency. As mentioned above, varying fiber length or diameter influences EF threshold through altering effective sinks or sources created by applied EF, and their efficacy is correlated to somatic depolarization. In fact, once artificial model is activated by EFs, we can always record AP at the soma. Thus, the AP latency in soma has little effects on computed EF threshold for activating CA1 pyramidal cells. However, such time delay is related to I-waves, since its duration usually covers the range of the first and second I-waves^55^. Thus, somatic AP latency is a potential factor that contributes to the variable physiological response to magnetic or electric field stimulus.

There are several limitations of the model and technical considerations in our study. First, our study addresses the importance of cell morphology in determining EF firing threshold. In fact, the transfer function of single neuron can be mediated by many factors, such as current kinetics^28, 29, 44, 46, 47^, dendritic integration^37, 38^, shunting inhibition^46, 47, 56^, autaptic transmission^57, 58^, neuronal noise^53, 59^, and so on. Understanding how cell morphology participates in field-induced response in the presence of these factors will surely facilitate our interpretation of variable neural response to EFs. Second, the basic morphologies of soma, axon and dendrites are varied separately in our simulation. Future work will focus on how possible combinations of these morphologies affect EF threshold for activating pyramidal cells. Finally, our simulation is performed at the cellular level. One goal of rational electromagnetic stimulation is to improve cognitive function with minimal disruption of brain network activity^7, 60^. Investigating how applied fields modulate brain network dynamics and how cell morphology contributes to such modulations are both necessary steps toward this goal, which should also be considered in following studies.

To conclude, the EF applied to neurons is a typical distributed stimulus. Its effect depends on the morphology and biophysics of the cell, the intensity of the field, and the orientation of neural element relative to EF^5–7, 26–33^. Such spatial and temporal interactions make it difficult to predict and interpret variable response to EFs at the cellular level. Central to addressing the unpredictable effects of field stimulation is the notion of “computational neurostimulation”, where interventional strategies are informed by quantitative models^61^. The present study with biophysical models addresses the significance of cell morphology in the modulatory effects induced by subthreshold and suprathreshold fields. It is a pivotal first step toward probing the degree and type of EF stimulation, which is helpful to better understand the nature of its action and how applied field activates neural elements. Further, our predictions also contribute to interpreting how the dose of noninvasive brain modulation strategies (such as TMS or tDCS) affects neural coding and network activity, which thus may facilitate the rational design of relevant devices and stimulation protocols.

## Methods

### Pyramidal neuron model

**Figure 9:**
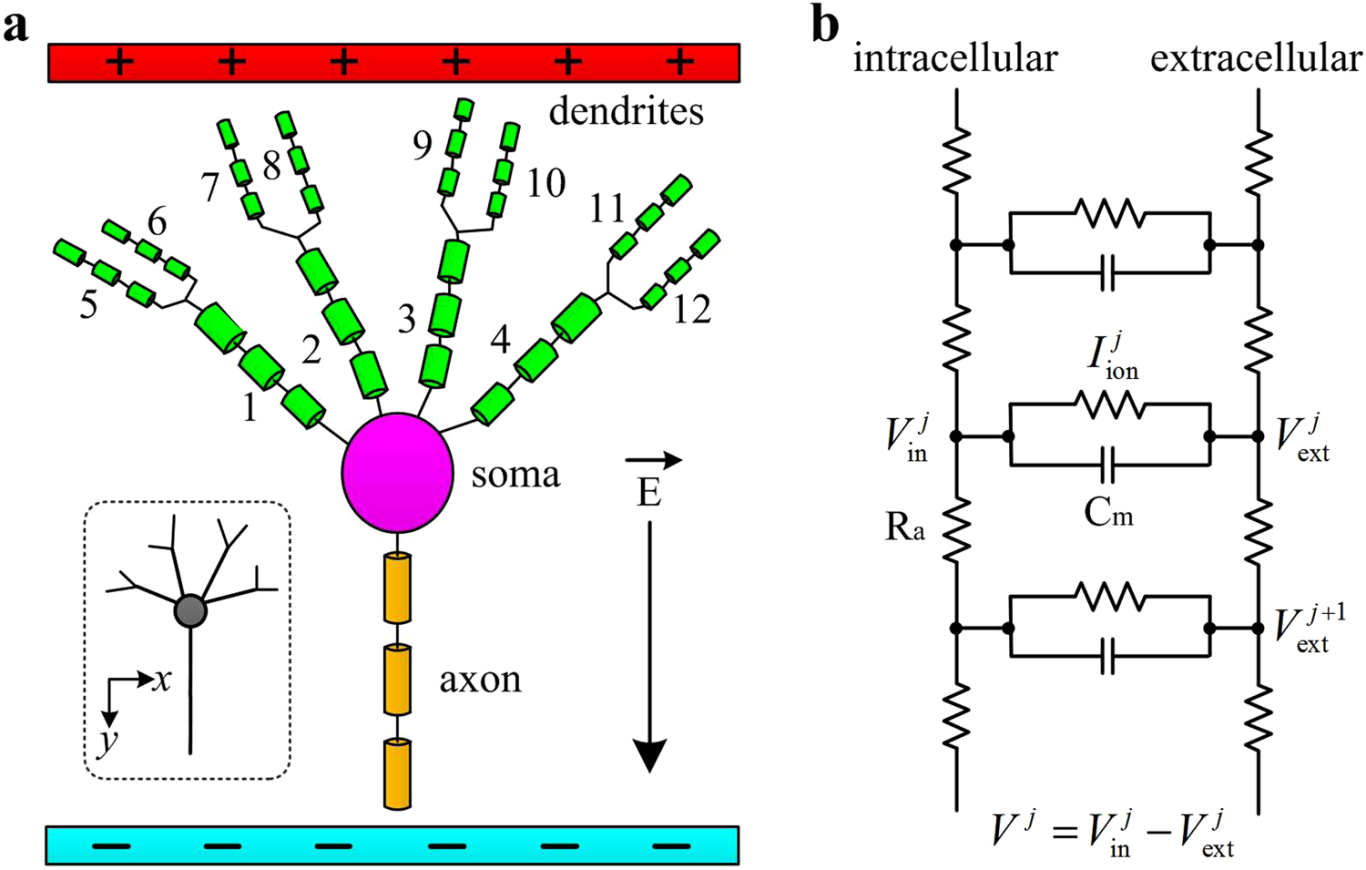
Artificial pyramidal neuron. (**a**) Schematic diagram of the multi-compartment artificial model stimulated by uniform EF parallel to the somato-dendritic axis (i.e., *y* axis). Positive EF is created by placing a negative electrode close to the axon and a positive electrode close to the dendrites. (**b**) Circuit representation of the active compartments.

The schematic view of the artificial model is shown in Fig. 9a, which consists of a soma, an axon and several dendrites. This artificial neuron is divided into a sequence of compartments in NEURON. The neighbor compartments are linked by axial resistivity R_a_ (Fig. 9b), which allows APs to propagate between neuronal segments. The initial length is 10 μm for soma, 50 μm for axon, 8 μm for No. 1–4 proximal dendrites, and 2 μm for No. 5–12 distal dendrites. The initial diameters for soma, axon and dendrite are 10 μm, 2 μm, and 1 μm, respectively. Note that a pyramidal neuron is so named because it has several basal dendrites that form a pyramid and an apical dendrite that emanates from the tip of the pyramid. Such complex dendritic topologies are missing in our artificial neuron. Here we only focus on the basic structures commonly found in the tree-like topology of dendrites, such as number, length, diameter, bend and branch.

The spatial structure of the artificial neuron is modeled by the cable equation in NEURON^36^

1 $${{\rm{C}}}_{{\rm{m}}}\frac{\partial V}{\partial t}+{I}_{{\rm{ion}}}(x,t)=\frac{{\rm{d}}}{4{{\rm{R}}}_{{\rm{a}}}}\frac{{\partial }^{2}V}{\partial {x}^{2}}$$

here *V* is the transmembrane potential, which is the difference between intracellular potential *V*_in_ and extracellular potential *V*_ext_, i.e., *V* = *V*_in_−*V*_ext_. Since most cable theory studies neglect the effects of extracellular potential^36, 62^, the *V*_ext_ in the absence of EF stimulation is set to be constant, i.e., 0 mV. C_m_, d, R_a_ are respectively membrane capacitance, diameter and cytoplasm resistivity of the fiber. *I*_ion_(*x, t*) is the sum of active and leakage currents on cell membrane. The integration method in NEURON is to discretize cable equation (1) into a multi-compartmental model. The discretization involves two approximations^36^. One is that the spatially-varying ionic currents are represented by their values at the center of each compartment. The other one is that the axial current is determined by the voltage difference between the center points of neighbor compartments. Then, the cable equation (1) is discretized into the following form

2 $${{\rm{C}}}_{{\rm{m}}}\frac{{\rm{d}}{V}^{j}}{{\rm{d}}t}+{I}_{{\rm{ion}}}^{j}({V}^{j},t)=\frac{{\rm{d}}}{4{{\rm{R}}}_{{\rm{a}}}}\frac{{V}^{j+1}-2{V}^{j}+{V}^{j-1}}{{\rm{\Delta }}{x}^{2}}$$

where *V* ^*j*^ is the membrane potential of compartment *j*, Δ*x* is the length of the compartment, and ${I}_{{\rm{ion}}}^{j}({V}^{j},t)$ is the total ionic current flowing through the cell membrane of compartment *j*.

The biophysical properties of the artificial neuron follow the descriptions by Berzhanskaya *et al*.^9^, which is based on the minor modifications of CA1 pyramidal model proposed and validated by Migliore *et al*.^63, 64^. The relevant simulation files are available for public download under the ModelDB section of the Senselab database (http://senselab.yale.med.edu). The active ionic channels with voltage-dependent activation include inward transient Na^+^ current (*I*_Na_), outward delayed rectifier K^+^ current (*I*_Kdr_), rapidly inactivating K^+^ current (*I*_Ka_), and non-specific hyperpolarizing cation current (*I*_h_). The conductance densities for *I*_Na_ and *I*_Kdr_ are both fixed in the whole model, which are 200 pS/μm^2^ and 100 pS/μm^2^. For *I*_Ka_, its conductance density in soma is 250 pS/μm^2^, and increases linearly with the distance from soma by 250 pS/μm^2^ per 100 μm in dendrites. Similar to *I*_Ka_, the conductance density for *I*_h_ in dendritic chambers is increased from 0.5 pS/μm^2^ in soma by 1.5 pS/μm^2^ per 100 μm. For the whole model, membrane capacitance is ${{\rm{C}}}_{{\rm{m}}}=1{\rm{\mu }}{\rm{F}}/{{\rm{cm}}}^{{\rm{2}}}$, axial resistance is R_a_ = 80Ω cm, and membrane resistance is R_m_ = 28 KΩ cm^2^. Other parameters in artificial neuron are identical with those described by Berzhanskaya *et al*.^9^.

Except above artificial neuron, we also examine the response of three complex cells with realistic morphologies to EFs. They are respectively the rat hippocampal CA1 pyramidal neuron, the L4 spiny neuron in cat visual cortex, and the interneuron in rat dorsal lateral geniculate nucleus. The biophysical and morphological data for these neurons are all available for public download under the ModelDB section of the Senselab database (http://senselab.yale.med.edu).

### Electric field introduction

Uniform EF stimulation with orientation parallel to the somatic-dendritic axis is applied to each model neuron. We use the extracellular mechanism of NEURON to describe the interactions between EF and cellular activity^9, 36, 62^. The EF stimulation is modeled by introducing an additive extracellular potential to each segment of the neuron. We take the cathode as the reference ground potential. Simultaneously, the horizontal and vertical axes are respectively denoted as *x* and *y* axes (Fig. 9a). For an applied field *E* with arbitrary direction, its *x*-component is ${E}_{x}=E\,\sin \,{\rm{\phi }}$ and *y*-component is ${E}_{y}=E\,\cos \,{\rm{\phi }}$, where φ is the angle between EF and *y* axis. In our simulation, we only focus on the effects of cell morphologies on EF-evoked responses and do not alter field direction. Thus, the value of φ is set to zero.

In NEURON simulation environment, the membrane potential for any compartment is approximated by using the voltage of its middle point. In the presence of uniform EF parallel to *y* axis, the potential of each extracellular node *j* (i.e., ${V}_{{\rm{ext}}}^{j}$) can be defined by

3 $${V}_{{\rm{ext}}}^{j}=Ed$$

where *d* is the distance of node *j* from cathode. In this case, the extracellular potential decreases linearly along the *y* axis while remains constant along the *x* axis. Note that the impact of *z* axis is omitted here, since there is no field component in that direction. According to equation (3), the extracellular potential difference between compartment *j* and *j* + 1 can be expressed by^9^

4 $${V}_{{\rm{ext}}}^{j+1}-{V}_{{\rm{ext}}}^{j}=E{\rm{\Delta }}l\,\cos \,{\rm{\gamma }}$$

here Δ*l* is the distance between two adjacent compartments, and γ is the angle between neuronal element and *y* axis.

Previous experiment^65^ has shown that neurons are insensitive to transverse field stimulation relative to axial stimulation. Then, the charge movement directly induced by EF in a transverse direction (i.e., across cell membrane) is usually negligible compared to the axial current flow induced by applied field^33^. Based on this assumption, we can explain equation (4) more simply as follows. If the dendrite, axon or soma is parallel to the field lines (i.e., the *y* axis), the spatial gradient in extracellular membrane potential and the field-induced current flow in the axial direction are both maximized. If it is perpendicular to the *y* axis, there is no induced current flowing in the axial direction. Further, the potential difference between two electrodes also induces additive current flow through the extracellular media. But it is neglected here and we only consider the effects of potential difference caused by uniform EF between neighboring extracellular compartments.
